# Degradable Polyampholytes from Radical Ring-Opening Copolymerization Enhance Cellular Cryopreservation

**DOI:** 10.1021/acsmacrolett.2c00298

**Published:** 2022-06-29

**Authors:** Théo Pesenti, Chen Zhu, Natalia Gonzalez-Martinez, Ruben M. F. Tomás, Matthew I. Gibson, Julien Nicolas

**Affiliations:** † Université Paris-Saclay, CNRS, Institut Galien Paris-Saclay, 92296 Châtenay-Malabry, France; ‡ Department of Chemistry, University of Warwick, Gibbet Hill Road, CV4 7AL, Coventry, U.K.; § Division of Biomedical Sciences, Warwick Medical School, University of Warwick, Gibbet Hill Road, CV4 7AL, Coventry, U.K.

## Abstract

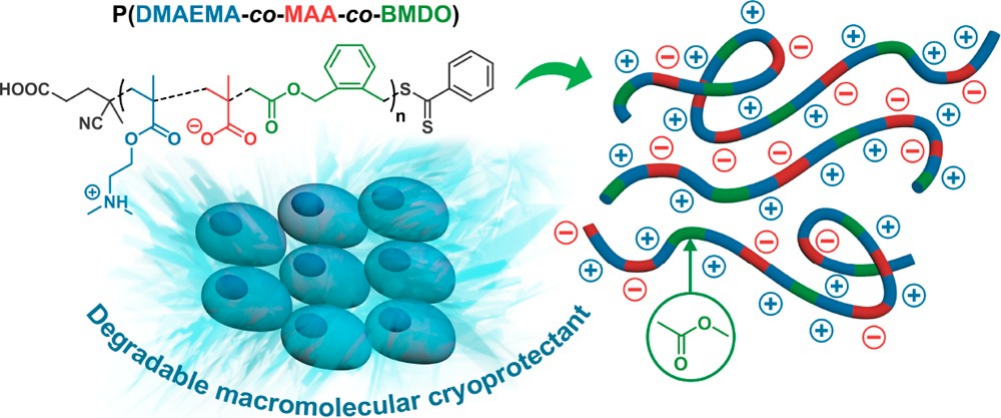

Macromolecular cryoprotectants based on polyampholytes are showing promise as supplemental cryoprotectants alongside conventional DMSO-based freezing. Here we exploit radical ring-opening (ter)polymerization to access ester-containing cryoprotective polyampholytes, which were shown to be degradable. Using a challenging cell monolayer cryopreservation model, the degradable polyampholytes were found to enhance post-thaw recovery when supplemented into DMSO. This demonstrates that degradable macromolecular cryoprotectants can be developed for application in biotechnology and biomedicine.

Macromolecular cryoprotectants^1^ are an emerging class of polymeric materials capable of mitigating damage to biological materials (cells, proteins, viruses) associated with the cold stress during cryopreservation. Current cryopreservation strategies based on DMSO do not always lead to full recovery. Not all cell types or formats (e.g., 2/3-D cell models^2,3^) can be efficiently stored, or are incompatible,^4^ with DMSO alone. There are also concerns of DMSO cytotoxicity, so strategies to reduce or remove it are required.^5−7^ Several classes of macromolecular cryoprotectants are emerging, including those inspired by, or mimicking, ice binding proteins^8−11^ such as poly(vinyl alcohol),^12^ alanine/lysine copolypeptides,^13^ graphenics,^14^ and cyclic peptides.^15,16^ These materials have been used to improve the cryopreservation of various cell types,^17−20^ bacteria,^21^ and proteins.^22,23^ Matsumura and co-workers have introduced polyampholytes (i.e., polymers with mixed cationic/anionic side chains) as potent macromolecular cryoprotectants.^24^ Polyampholytes have proven to lead to large increases in cell recovery post-thaw in both slow freezing and vitrification across a range of cell types.^25−28^ The mechanism of action of polyampholytes is not fully understood,^29^ but they only have weak effects on ice growth.^30^

A key challenge in the design and discovery of any biomaterial which could have *in vivo* use is the need for it to be removed from the body, by being sufficiently small to be excreted, or by using degradable polymers. Dextran-based (a polysaccharide) polyampholytes are (to the best of our knowledge) the only potentially degradable polyampholytes reported for cryopreservation.^31^ Biomaterials derived from controlled radical polymerization are appealing due to the wide range of potential monomers and opportunities for precision macromolecular engineering. However, a drawback is that their carbon–carbon backbones are not degradable. Cyclic ketene acetals (CKAs), under appropriate conditions, undergo radical ring-opening polymerization (rROP), leading to main-chain esters being installed into a backbone that is otherwise derived from conventional vinyl monomers.^32,33^ In recent years,^34^ the development of rROP-based systems by copolymerization of CKAs with vinyl monomers has gained considerable momentum for a wide range of applications, including nanocarriers for drug delivery applications,^35,36^ bioconjugates,^37,38^ marine antibiofouling surfaces,^39^ and latex particles.^40^ Copolymerization of chloro-vinyl acetate with 5,6-benzo-2-methylene-1,3-dioxepane (BMDO) as a CKA allowed access to ice-binding poly(vinyl alcohol) with esters in the main chain for degradation.^41^ However, there are no reports of polyester-like polyampholytes, and hence the impact of such functionality on their cryoprotective function is unknown. Importantly, degradable polyampholytes would also enable macromolecular cryoprotectants to move from *ex vivo*/*in vitro* applications toward *in vivo*.

We herein incorporate ester linkages into polyampholytes using radical-ring-opening terpolymerization, allowing up to 15 mol % of ester backbone units to be introduced. To enable methacrylic acid units to be included, a synthetic strategy using *tert*-butyldimethylsilyl (TBDMS) protection was developed anticipating side reactions between BMDO and methacrylic acid (MAA), and allowing efficient deprotection under mild conditions. The copolymers are shown to be hydrolytically degradable in alkaline medium and to match the cryopreservation performance of a nondegradable polyampholyte counterpart.

To obtain a polyampholyte for cryopreservation, it is essential to have the correct balance of anionic/cationic units.^1,24^ Poly(dimethylaminoethyl methacrylate)-*co*-poly(methacrylic acid) P(DMAEMA-*co*-MAA) were chosen as the target copolymers based on previous reports that this pairing can lead to a cryoprotective outcome in suspension cryopreservation, although they have not been used in challenging monolayer cryopreservation (explored here).^42^ The synthesis of P(DMAEMA-*co*-CKA) copolymers has been successfully reported,^43−46^ but the copolymerization with MAA with CKA is not straightforward.^47^ Electrophilic addition of the carboxylic acid group of MAA onto the C=C double bond of the CKA indeed results in the formation of two distinct copolymers (depending on the initial monomer feed ratio), which lack either the pendant carboxylic acids or the ester bonds in the backbone. Consequently, a protected MAA is required. From initial considerations the use of methyl methacrylate (MMA) could appear as a suitable candidate, especially considering the already reported synthesis of P(DMAEMA-*co*-MMA-*co*-MDO) terpolymer.^48^ However, we suspected that the demethylation conditions would be too harsh to allow deprotection without cleavage of the ester backbone, as recently hypothesized in the acid-mediated deprotection of poly(2-methylene-1,3-dioxepane*-co*-*tert-*butyl acrylate) copolymers.^49^ We therefore used *tert*-butyldimethylsilyl methacrylic acid (TBDMSMA) as a protected methacrylic acid,^50^ whose deprotection required milder conditions (Figure 1) to which a main-chain ester would be stable. Silyl ethers have already been used as hydroxyl protecting groups for CKA-based polymers and have been easily deprotected by fluorine anions (F^–^).^51^ On the other hand, the copolymerization of CKA with silylated methacrylates has also been reported, but never subjected to deprotection.^52^

**Figure 1:**
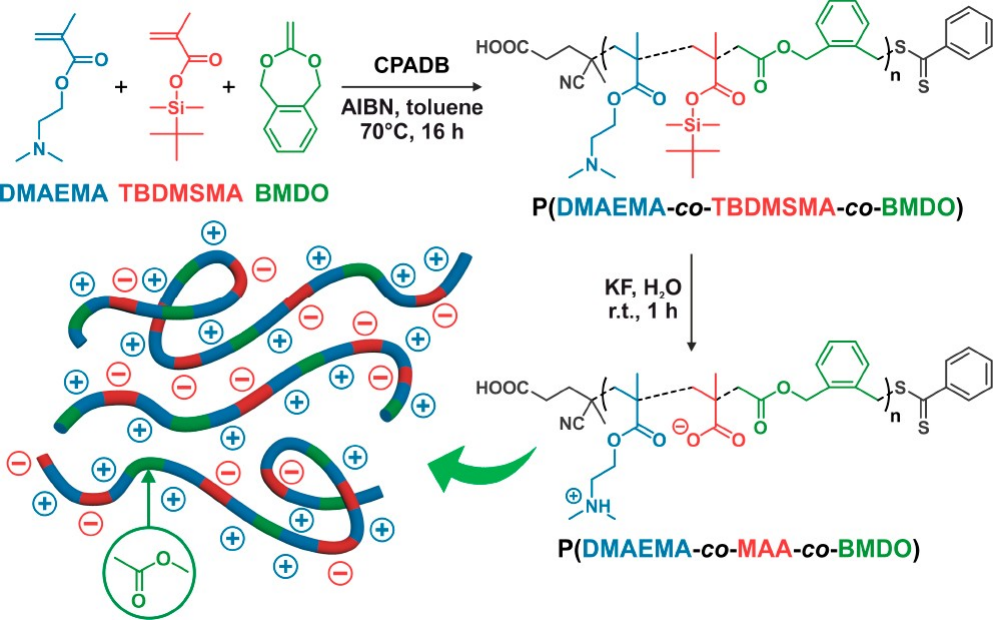
Synthesis of poly(*N,N*-dimethylaminoethyl methacrylate-*co*-methacrylic acid-*co*-5,6-benzo-2-methylene-1,3-dioxepane) P(DMAEMA-*co*-MAA-*co*-BMDO) terpolymers by RAFT terpolymerization of DMAEMA, TBDMSMA, and BMDO with CPADB as a RAFT agent, followed by selective KF-mediated deprotection of TBDMSMA units.

The RAFT terpolymerization conditions were based on those for the homopolymerization of TBDMSMA.^50^ 4-Cyano-4-(phenylcarbonothioylthio)pentanoic acid (CPADB) was used as the RAFT agent at 70 °C for 16 h in anhydrous toluene. Three different P(DMAEMA-*co*-TBDMSMA-*co*-BMDO) terpolymers were synthesized by varying the initial BMDO content (**P0-TBDMS**, **P1-TBDMS**, and **P2-TBDMS** for *f*_BMDO,0_ = 0, 0.375 and 0.5, respectively), while keeping a TBDMSMA:DMAEMA molar ratio of 2:3 (Table S1). *M*_n_ values were in the 7100–8600 g·mol^–1^ range with fairly low dispersities (*Đ* = 1.33–1.43). The ^1^H NMR spectra (Figure 2a) showed the characteristic proton signals from DMAEMA (protons *i*, *j* and *k*) and TBDMSMA units (protons *h* and *g*). The ^1^H NMR spectra of **P1-TBDMS** and **P2-TBDMS** also exhibited characteristic proton signals from BMDO (protons *b*, *c*, and *c’*), thus confirming the formation of the expected terpolymers.

**Figure 2:**
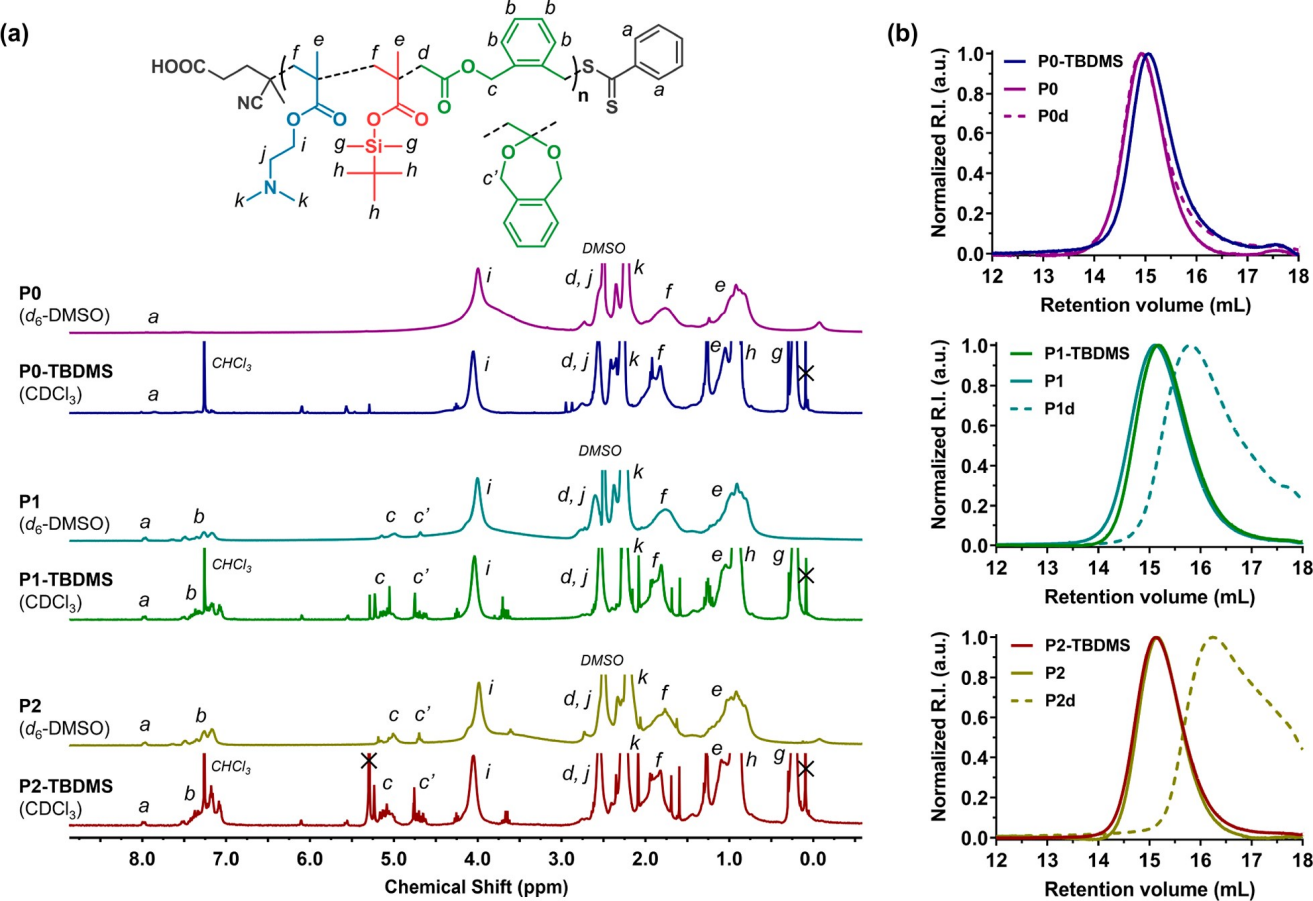
(a) ^1^H NMR spectra in CDCl_3_ or *d*_6_-DMSO of **P0-TBDMS**, **P1-TBDMS**, **P2-TBDMS**, **P0**, **P1**, and **P2** terpolymers. (b) SEC chromatograms (DMSO with 0.1 M LiBr, PMMA calibration) of **P0-TBDMS**, **P1-TBDMS**, **P2-TBDMS**, **P0**, **P1**, and **P2** terpolymers, as well as their degradation products (**P0d**, **P1d**, **P2d**, respectively).

Deprotection of terpolymers **P0-TBDMS**, **P1-TBDMS**, and **P2-TBDMS** (into **P0**, **P1**, and **P2**, respectively) was first attempted using TBAF in THF for 1 h at room temperature. Purification was performed either by precipitation into methanol or by dialysis in THF and water. However, purification was not successful, as remaining tetra-*n-*butylammonium (TBA) signals were still observed by ^1^H NMR, likely because TBA cations might act as a counterion of some negatively charged carboxylates. An alternative deprotection route was therefore employed, using aqueous KF as the deprotecting agent for 1 h at room temperature, followed by purification by dialysis in water. By ^1^H NMR (Figure 2a), TBDMS-related signals at δ = 0.96 and 0.25 ppm disappeared while all the other signals remained. The efficient removal of fluorinated byproducts was confirmed by ^19^F NMR analysis (Figure S1). SEC analyses did not show any significant shift toward lower *M*_n_ values, thus ruling out degradation of the terpolymer backbone during deprotection (Figure 2b). Conversely, a slight shift toward higher *M*_n_ values was observed upon deprotection, which is likely related to the formation of carboxylic acid groups that may impact the conformation of terpolymer chains and thus their elution. This confirmed the formation of **P0**–**P2** (*M*_n_ = 8 200–11 300 g·mol^–1^ and *Đ* = 1.24–1.41), with **P1** and **P2** exhibiting ∼85% open BMDO units and *F*_BMDO_ = 0.12 and 0.15, respectively.

Hydrolytic degradation of **P1** and **P2** terpolymers was then performed under accelerated conditions (2.5 wt % KOH in DMSO/MeOH 1:1 (v/v)) for 1 day at room temperature to demonstrate that these polymers could be degraded. Significant shifts toward lower molar masses were observed (Figure 2b), thus confirming the successful insertion of open BMDO units into the terpolymers. The *M*_n_ loss increased with the *F*_BMDO_ value as −73% and −82% decrease in *M*_n_ were measured for *F*_BMDO_ = 0.12 and 0.15, respectively, which were in rather good agreement with the theoretical values (Table 1). As expected, the CKA-free copolymer (**P0**) did not show any degradation after 1 day under the same conditions, which was evidenced by the perfect overlay of the two SEC traces.

**Table 1:** Macromolecular Characteristics of P(DMAEMA-*co*-MAA-*co*-BMDO) Terpolymers Synthesized by RAFT Polymerization of DMAEMA, TBDMSMA (and BMDO) at 70 °C for 16 h at [M] = 1.5 mol·L^–1^ in Anhydrous Toluene with M = All Monomers and [M]:[CPADB]:[AIBN] = 100:1:0.2

Entry	*F*_DMAEMA_:*F*_MAA_:*F*_BMDO_^a^	open BMDO (%)^b^	*M*_n, exp_ (g mol^–1^)^c^	*Đ*^c^	theo *M*_n, deg_ (g mol^–1^)^d^/theo *M*_n_ loss (%)^e^	exp *M*_n, deg_ (g mol^–1^)^c^/exp *M*_n_ loss (%)^f^
**P0**	0.67:0.33:0	–	11 300	1.24	–	–/–
**P1**	0.52:0.36:0.12	84	8200	1.41	1300/–83%	2100/–73%
**P2**	0.48:0.37:0.15	85	8300	1.24	1000/–88%	1500/–82%

a Determined by ^1^H NMR, by integrating 6H from Si-CH_3_ groups (0–0.35 ppm), 2H of DMAEMA (4 ppm), 2H of open BMDO (5.0–5.2 ppm), and 4H of closed BMDO (4.6–4.8 ppm).

b Determined by ^1^H NMR by integrating 2H of open BMDO (5.0–5.2 ppm) and 4H of closed BMDO from (4.6–4.8 ppm).

c Determined by SEC (DMSO with 0.1 M LiBr, PMMA calibration).

d Determined according to theo *M*_n, deg_ = [1/(open BMDO × *F*_BMDO_) – 1] × [(*F*_DMAEMA_ × MW_DMAEMA_ + *F*_MAA_ × MW_MAA_)/(*F*_DMAEMA_ + *F*_MAA_)] + MW_BMDO_ with MW being the molecular weight of the considered monomer.

e Determined according to theo *M*_n_ loss = (theo *M*_n, deg_ – *M*_n, exp_)/*M*_n, exp_.

f Determined according to exp *M*_n_ loss = (exp *M*_n,deg_ – *M*_n, exp_)/*M*_n, exp_.

With successful demonstration of the synthesis of a degradable polyampholyte, the proof-of-principle for cryopreservation could be undertaken. A549 (lung epithelial adenocarcinoma) cell monolayers were selected, as they are a commonly used cell line, but have been previously explored in monolayer cryopreservation, allowing comparison to previous studies.^20,26^ It is important at this point to stress that the cryopreservation of cells in monolayer format is significantly more challenging than that in suspension, as, in addition to cell death, cell detachment can reduce the post-thaw yield.^53^ The cytotoxicity of **P1** was evaluated by a resazurin (metabolic assay) following 24 h incubation (Figure S2, Supporting Information). As expected for this class of copolymer, there was a reduction in cell viability to ∼50% at higher (20 mg·mL^–1^) concentrations. This has previous been observed for polyampholytes^26,54^ and does not limit their use in cryopreservation. For cryopreservation the cells are only exposed to the cryoprotectant copolymer for a very short period of time (<30 min) before freezing, and upon thawing is washed away. Hence, although cytotoxicity information is important it does not exclude application. To evaluate cryopreservation, 20 mg·mL^–1^ copolymer in 10% (v/v) DMSO was applied to A549 monolayers for 10 min, then excess media was removed, and the cells were directly frozen and stored at −80 °C. After 24 h, the cells were thawed and allowed to recover for 24 h before total cell recovery was measured (Figure 3a). This latter point is essential, as shorter post-thaw times and only measuring viability (not cell yield) have been shown to overestimate the potency of cryoprotectants and give false positives.^55^ With DMSO, the post-thaw cell yield was approximately 50%, which is higher than previously reported^26^ due to the use of optimized freezing conditions (Figure 3b). Addition of **P1** led to an 80.3% post-thaw cell yield, demonstrating a significant enhancement. A positive control of a nondegradable polyampholyte ((poly(vinyl ether-*alt*-maleic acid mono(dimethylamino ethyl)ester))), **polyampholyte-1**(26) gave 89.3% recovery. Such an apparently very small decrease in performance of **P1** compared to **polymampholyte-1** in terms of cell recovery was however not statistically significant, which shows that conferring degradability to P(DMAEMA-*co*-MAA) was not at the expense of its cryoprotective ability. Cell viability measurements agree with the cell recovery, showing ∼90% viability for **P1**, which is also similar to that of **polyampholyte-1** (Figure 3c). Finally, imaging of the cells confirms a higher surface coverage post-thaw with the polyampholytes, compared to DMSO alone (Figure 3d).

**Figure 3:**
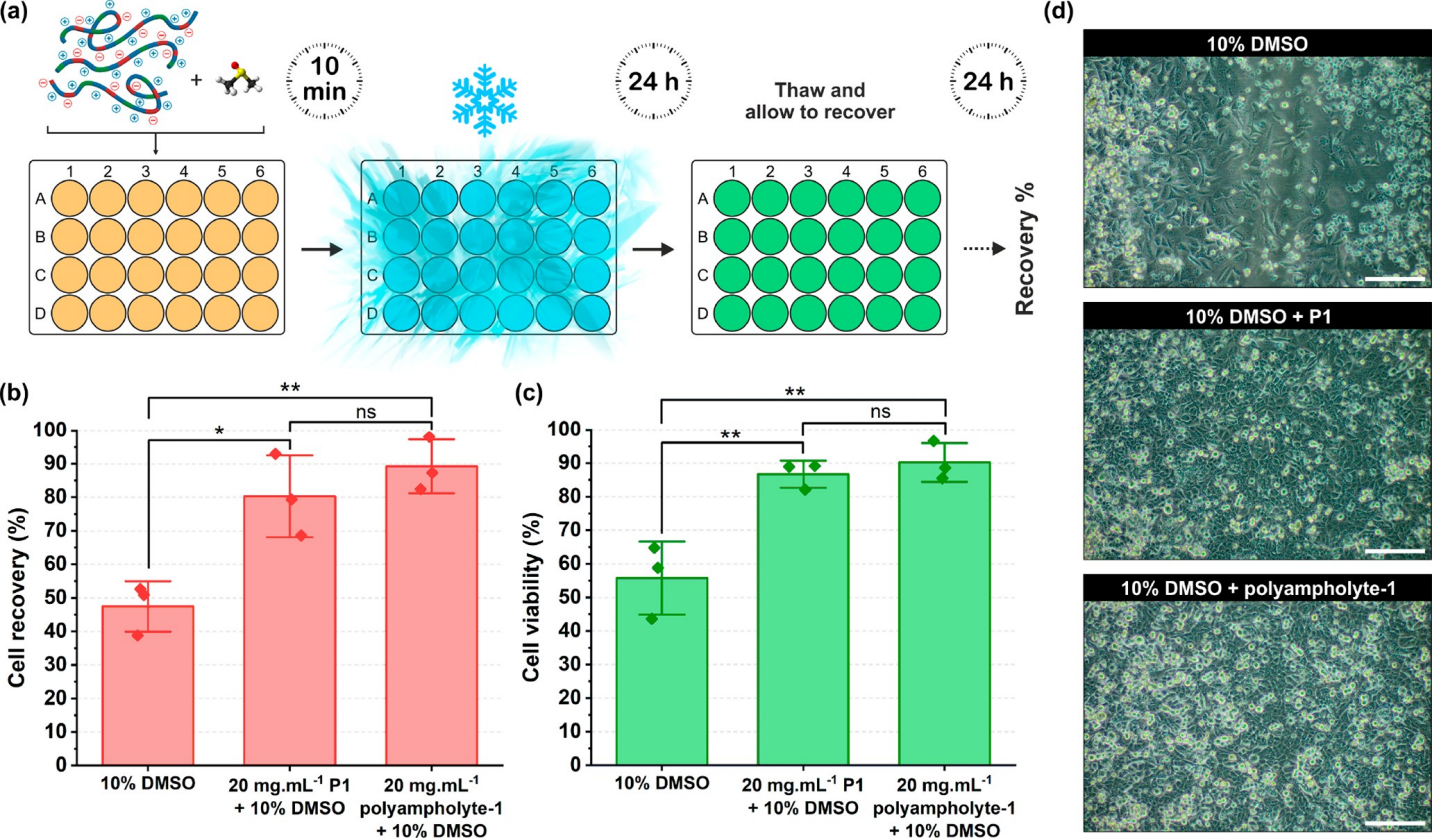
A549 cell monolayer cryopreservation. (a) Schematic of the monolayer cryopreservation and post-thaw process. (b) Cell recovery 24 h post-thaw, relative to prefreezing, determined using trypan blue exclusion test. (c) Cell viability 24 h post thaw determined using trypan blue exclusion test. (d) Phase contrast microscopy of A549 cells 24 h post-thaw. Scale bar indicates 200 μm. Results expressed as mean ± SD (*n* = 3 for each condition). One-way ANOVA with Tukey’s posthoc test. * = *P* < 0.05, ** = *P* < 0.001 considered as statistically significant different using a 95% confidence level, ns = not significant.

Macromolecular cryoprotectants have the potential to revolutionize cellular cryopreservation, but for many biomedical applications degradable materials will be essential. Here we introduce the use of rROP to insert ester units into polyampholytes introducing degradability, while allowing the use of conventional vinyl-based monomers and controlled radical polymerization. To enable the incorporation of methacrylic acid (as anionic component) *tert*-butyldimethylsilyl protecting groups were used, ensuring chemo-selective deprotection, without significant *M*_n_ loss which is an important improvement in comparison to deprotection conditions requiring acidic conditions.^49^ These new polyampholytes were shown to be noncytotoxic under conditions relevant for cryopreservation. The polyampholytes were shown to significantly increase the post-thaw cell yield, and cell viability, of a challenging cell monolayer cryopreservation model demonstrating that the dilution of the charged monomer units with the esters did not remove the cryoprotectant activity. These results are important, as they show that polyampholytes can be designed and synthesized which may be suitable for *in vivo* usage as components to protect emerging cell-based therapies from damage during cold chain handling.

## Supporting Information Available

Full synthetic and characterization details as well as additional cytotoxicity testing are included. (PDF)
