# Supplementary material for: Degradable Polyampholytes from Radical Ring-Opening Copolymerization Enhance Cellular Cryopreservation
Source: ACS Macro Lett. 2022 Jun 29;11(7):889–94. doi: 10.1021/acsmacrolett.2c00298 (PMC9301905; doi:10.1021/acsmacrolett.2c00298)
Supplement: Supplementary file 1 — mz2c00298_si_001.pdf [file mz2c00298_si_001.pdf]

# Supporting Information For

Degradable Polyampholytes from Radical Ring-Opening Copolymerization Enhance Cellular Cryopreservation

Théo Pesenti,[a] Chen Zhu,[a] Natalia Gonzalez-Martinez[b,c] Ruben M. F. Tomás,[b] Matthew I. Gibson[b, c,*] and Julien Nicolas[a,*]

[a] Université Paris-Saclay, CNRS, Institut Galien Paris-Saclay, 92296 Châtenay-Malabry, France;

[b] Department of Chemistry, University of Warwick, Gibbet Hill Road, CV4 7AL, Coventry, UK;

[c] Division of Biomedical Sciences, Warwick Medical School, University of Warwick, Gibbet Hill Road, CV4 7AL, Coventry, UK.

*To whom correspondence should be addressed.

MIG (Email: M.I.Gibson@warwick.ac.uk; Tel.: (+44) 0247 65 24803)

JN (Email: julien.nicolas@u-psud.fr; Tel.: (+33) 1 46 83 58 53)

## Experimental section

### Materials

Azobisisobutyronitrile (AIBN) was purchased from Sigma-Aldrich and recrystallized from MeOH. 2-(Dimethylamino) ethyl methacrylate (DMAEMA) was purchased from Sigma-Aldrich and purified through a small pad of basic alumina before use. 4-Cyano-4-(phenylcarbonothioylthio)pentanoic acid (CPADB) was purchased from Sigma-Aldrich and used as received. Tetrabutylammonium fluoride (TBAF) at 1.0 M in THF was purchased from Carl Roth and used as received. *Tert*-butyldimethylsilyl methacrylate (TBDMSMA)[1] and 5,6-benzo-2-methylene-1,3-dioxepane (BMDO)[2] were synthesized as described elsewhere. All other reagents were purchased from Merck, TCI Chemicals or Thermo Fischer. Deuterated solvents and anhydrous solvents were purchased from Eurisotop and Merck, respectively. All other solvents were purchased from Carlo Erba at the highest grade.

### Analytical Methods

*Nuclear Magnetic Resonance Spectroscopy (NMR).* NMR spectroscopy was performed in 5 mm diameter tubes in $CDCl_3$ or $d_6$-DMSO at 25 °C. $^1$H NMR spectroscopy was performed on a Bruker Avance spectrometer at 300 MHz. $^{19}$F NMR spectra were recorded on a Bruker Avance spectrometer at 200 MHz. The chemical shifts are reported in ppm ($\delta$ units), and internal solvent signal ($\delta$ = 7.26 ppm for $CDCl_3$, $\delta$ = 2.5 ppm for $d_6$-DMSO) was used as reference.

*Size Exclusion Chromatography (SEC).* SEC was performed on a Viscotek TDA 302/GPCmax from Malvern with a differential refractive index detector, low and right-angle light scattering detectors and a differential viscometer detector, and two columns in series from Agilent (PL PolarGel-M, 300 × 7.5 mm; bead diameter 8 µm; molar mass range 1 000–500 000 g.mol$^{-1}$)

preceded by a guard column from Agilent Technologies (PL PolarGel-M, 7.5 × 50 mm; bead diameter 8 µm). Analyses were performed at 60 °C in DMSO (HPLC grade) with 10 mM LiBr (filtered over 0.22-µm PTFE filters) at a flow rate of 0.7 $mL.min^{-1}$. 2,6-Di-*tert*-butyl-4-methylphenol (BHT) (0.3 wt.%) was used as flow rate marker. All samples were filtered over 0.22 µm PTFE filters prior to injection. The calibration curve was based on poly(methyl methacrylate) (PMMA) (range 540–342 900 $g.mol^{-1}$) from Agilent. The OmniSEC software enabled the determination of $M_n$ (number-average molar mass), $M_w$ (weight-average molar mass), and $M_w/M_n$ (dispersity, $Đ$).

**Copolymer and terpolymer syntheses**

*Synthesis of poly(N,N-dimethylaminoethyl methacrylate-co-tert-butyl-dimethylsilyl methacrylate-co-5,6-benzo-2-methylene-1,3-dioxepane) P(DMAEMA-co-TBDMSMA-co-BMDO) (**P0-TBDMS**, **P1-TBDMS, P2-TBDMS**).* A typical procedure (**P0-TBDMS**) is as follows: in a 40 mL vial, fitted with a rubber septum and a magnetic stirring bar, AIBN (0.04 eq., $4.9\times10^{-3}$ mmol, 0.81 mg), CPADB (0.2 eq., $2.5\times10^{-2}$ mmol, 6.9 mg), DMAEMA (6 eq., 1.5 mmol, 232.9 mg) and TBDMSMA (4 eq., 1 mmol, 197.9 mg) were dissolved in anhydrous toluene (1.43 g; [M] = 1.5 $mol.L^{-1}$; [M]:[CTA]:[AIBN] = 100:1:0.2). The solution was bubbled with dry argon to remove dissolved oxygen for 15 min at room temperature and then immersed in a preheated oil bath at 70 °C for 16 h. The solution was then rapidly cooled under air. The reaction solvent was removed under reduced pressure and copolymer was then precipitated twice in cold hexane. The resulting **P0-TBDMS** copolymer was dried under high vacuum until constant weight to give a pink solid.

The same procedure was adapted as follows to synthesize P(DMAEMA-*co*-TBDMSMA-*co*-BMDO) terpolymers: **P1-TBDMS** [$f_{BMDO,0}$ = 0.375, AIBN (0.04 eq., $2.5\times10^{-2}$ mmol, 4.1 mg), CPADB (0.2 eq., $1.2\times10^{-1}$ mmol, 34.4 mg), BMDO (6 eq., 7.4 mmol, 1.20

g), DMAEMA (6 eq., 7.4 mmol, 1.16 g) and TBDMSMA (4 eq., 5 mmol, 0.99 g) were dissolved in anhydrous toluene (11.46 g; [M] = 1.5 mol.L$^{-1}$), [M]:[CTA]:[AIBN] = 200:1:0.2] and **P2-TBDMS** [$f_{BMDO,0}$ = 0.5, AIBN (0.04 eq., 2.5×10$^{-2}$ mmol, 4.1 mg), CPADB (0.2 eq., 1.2×10$^{-1}$ mmol, 34.4 mg), BMDO (10 eq., 0.012 mol, 2 g), DMAEMA (6 eq., 7.4 mmol, 1.16 g) and TBDMSMA (4 eq., 5 mmol, 0.99 g) were dissolved in anhydrous toluene (14.32 g; [M] = 1.5 mol.L$^{-1}$), [M]:[CTA]:[AIBN] = 200:1:0.2)]

*Synthesis of poly(N,N-dimethylaminoethyl methacrylate-co-methacrylic acid-co-5,6-benzo-2-methylene-1,3-dioxepane) P(DMAEMA-co-MAA-co-BMDO) (**P0**, **P1**, **P2**)*. In a glass vial, **P1-TBDMS** (789 mg, 0.9 mmol of TBDMSMA units, 1 equiv.) was mixed with 8 mL of a KF mixture (0.5 M, 2.5 equiv.). The reaction was left at room temperature for 1 h. The polymer was dialyzed (MWCO = 1 kDa, Biotech RC Spectra/Por®) against distilled water for 1 day then was freeze-dried to give **P1** as a reddish solid (400 mg). The same protocol was performed with **P2-TBDMS** (472 mg) to yield **P2** as a reddish solid (260 mg), and with **P0-TBDMS** (57 mg) to yield **P0** as a reddish solid (40 mg).

**Chemical degradation**

In a 7-mL vial, 7 mg of copolymer was dissolved in 350 µL of DMSO. Then, 350 µL of a KOH mixture (5 wt.% in MeOH) was added. The mixture immediately became yellowish and was left under magnetic stirring at room temperature for 1 day. 305 µL of HCl (1 M) was added to neutralize KOH. Solvents were evaporated under high vacuum followed by solubilization in DMSO (LiBr 0.1 M) and filtered over 0.22-µm PTFE syringe-filters prior SEC analysis. The resulting degraded copolymers were denoted **P0d, P1d** and **P2d** for **P0, P1** and **P2**, respectively.

**Table S1. Macromolecular characteristics of P(DMAEMA-*co*-TBDMSMA) copolymer and P(DMAEMA-*co*-TBDMSMA-*co*-BMDO) terpolymers synthesized by RAFT polymerization of DMAEMA, TBDMSMA (and BMDO) at 70 °C for 16 h at [M] = 1.5 mol.L$^{-1}$ in anhydrous toluene with M = all monomers and [M]:[CPADB]:[AIBN] = 100:1:0.2).**

| Entry | $f_{DMAEMA,0}$: $f_{TBDMSMA,0}$: $f_{BMDO,0}$ | $F_{DMAEMA}$: $F_{TBDMSMA}$: $F_{BMDO}$[a] | Open BMDO (%)[b] | Conv. DMAEMA (%)[c] | $M_{n,\,exp}$ (g.mol$^{-1}$)[d] | $Đ$[d] |
|---|---|---|---|---|---|---|
| **P0-TBDMS** | 0.6:0.4:0 | 0.68:0.32:0 | - | 76 | 8 600 | 1.38 |
| **P1-TBDMS** | 0.375:0.25:0.375 | 0.51:0.33:0.16 | 79 | 43 | 7 100 | 1.43 |
| **P2-TBDMS** | 0.30:0.20:0.50 | 0.46:0.32:0.22 | 79 | 32 | 8 000 | 1.33 |

[a] Molar fractions were determined by $^{1}$H NMR, by integrating: 6H from Si-$CH_3$ groups (0-0.35 ppm) for $F_{TBDMSMA}$, 2H of DMAEMA (4 ppm) for $F_{DMAEMA}$, and 2H of open BMDO (5.0–5.2 ppm) and 4H of closed BMDO (4.6–4.8 ppm) for $F_{BMDO}$. [b] Determined by $^{1}$H NMR by integrating the 2H of open BMDO (5.0–5.2 ppm) and 4H of closed BMDO from (4.6–4.8 ppm). [c] Determined by $^{1}$H NMR by integrating 2H of DMAEMA (5.6 and 6.2 ppm) and 2H at 4.0 ppm [d] Determined by SEC (DMSO with 0.1 M LiBr, PMMA calibration).

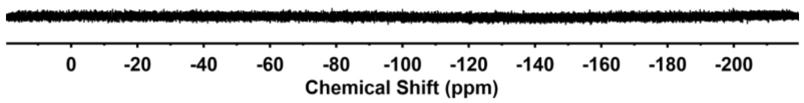


**Figure S1.** $^{19}$F NMR spectrum ($d_6$-DMSO) of **P1** from 0 to -200 ppm.

## Cell Culture

Adenocarcinomic human alveolar basal epithelial (A549) cells were cultured in 175 cm$^2$ cell culture flasks (Corning). The cell culture medium consisted of Ham's F12-K media (Gibco) supplemented with 10% foetal bovine serum (Sigma-Aldrich) and 1% antibiotic-antimycotic solution containing 10,000 units.mL$^{-1}$ of penicillin, 10 mg.mL$^{-1}$ streptomycin, and 25 µg.mL$^{-1}$ amphotericin B (Sigma-Aldrich). Cells were maintained at 37 °C and 5% $CO_2$ in an incubator

and passaged when they reached 80 % confluency, every 3-4 days. To passage cells, these were dissociated from the flask by treating them with 0.25 % Trypsin in EDTA (Gibco).

**Cell monolayer cryopreservation**

Methods were adapted from previous reports.[3,4] After dissociation, A549 cells were centrifuged at 2000 rpm for 5 minutes and resuspended using freezing media, which was supplemented with 10% FBS only. An aliquot of cells was taken, and the cell suspension was diluted 1:1 with 0.2 % trypan blue and counted using a haemocytometer (Sigma Aldrich)**.** Cells were seeded at 4 x $10^5$ cells per well in 24-well plates (Starlab) with 500 µL media. Cells were allowed to attach and form a one-cell thick monolayer in the incubator at 37 °C and 5 % $CO_2$. After 24 h, pre-freeze counts were performed for future cell recovery calculations. Media was removed from the plate and 500 µL of cryopreservation solution was added. The different cryopreservation conditions assessed (previously dissolved in freezing media) were 10% DMSO, 20 mg $mL^{-1}$ P1 in 10% DMSO, and 20 mg $mL^{-1}$ polyampholyte-1 in 10% DMSO. The solutions were previously sterile filtered using a 0.2 µm filter (Sartorius) and conditions were assessed in triplicates. After 10 minutes incubation, the cryoprotectant solutions were removed and the plate was transferred to a -80 °C freezer on top of a Corning CoolSink XT 96F plate. After 24 h, cells were thawed by adding 500 µL of warm cell culture media. The cells were placed in an incubator at 37 °C and 5% $CO_2$. 24 h post-thaw, cell counts were performed as previously described and percentage cell recovery was calculated by dividing the number of viable cells post-thaw by the number of cells frozen. For cell viability calculations, the supernatant from the post-thaw cell counting step was collected and cells present were counted as dead as they were not able to attach to the monolayer. Additionally, stained cells present in the monolayer were also counted as dead. This is expressed as formulae below. This analysis

is essential to ensure false positives from high viability, but low overall recovery are not reported.[4]

$$Recovery_{TB}\ (\%) = \frac{cells_{unstained}}{cells_{frozen}} \times\ 100 \qquad (2)$$

$$Viability_{TB}\ (\%) = \frac{cells_{unstained}}{cells_{unstained} + cells_{stained}} \times 100 \qquad (3)$$

**Polymer cytotoxicity assay**

Polymer cytotoxicity was tested by measuring the metabolic reduction of resazurin to resorufin as an indicator of cell viability after a 24-hour incubation period. A549 cells were seeded at 1 x $10^4$ cells per well in 100 µL of cell culture media in a 96-well plate with decreasing polymer concentrations from 20 to 0.625 mg $mL^{-1}$. Cells were incubated for 24 h in an incubator at 37°C and 5% $CO_2$. One tablet of resazurin sodium salt (Scientific Laboratory Supplies) was diluted 1 in 10 in cell culture media. After 24 h, cell culture media was discarded and 100 µL of the resazurin sodium salt mixture was added to all wells. Cells were incubated for 1-4 h at 37°C and 5% $CO_2$ and the absorbance was measured hourly using the Synergy HTX Multi-Mode Reader (BioTek) at 570 and 600 nm. Measurements were performed until the control cells reached approximately 80% resazurin reduction. Treated cells were compared to the untreated control cells and viability was expressed as a percentage.

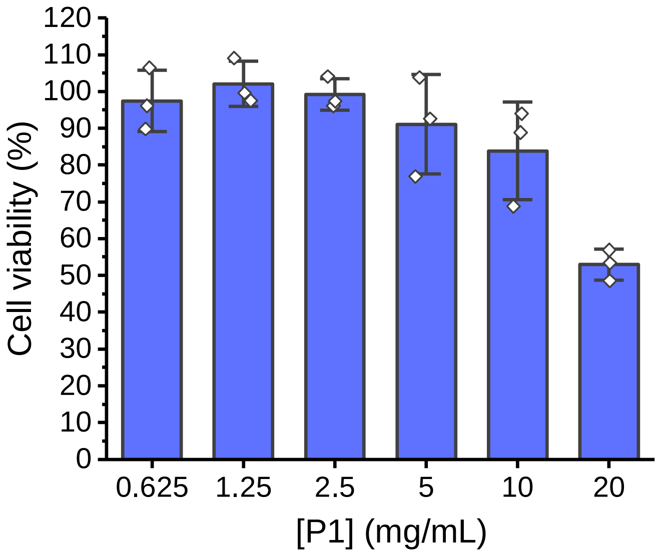


**Figure S2.** Cytotoxicity of **P1** as a function of molecular weight after 24-hour incubation. Viability was determined by a resazurin reduction assay and reported relative to an untreated control with no polymer. Results expressed as mean ± SD.

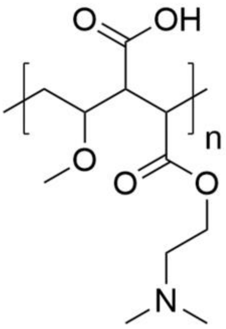


**Figure S3.** Chemical structure of the control polyampholyte used: poly(vinyl ether-*alt*-maleic acid mono(dimethylamino ethyl)ester); This will be referred to in text as **polyampholyte-1**.

**Statistical analysis**

One-way ANOVA and Tukey's post-hoc tests were performed using Origin (Version 2021). This was used to determine whether statistically significant differences in cell viability and recovery exist after cell monolayer treatment with different cryopreservation formulations. $P$ value $< 0.05$ was considered statistically significant.

## References


(1) Nguyen, M. N.; Bressy, C.; Margaillan, A. Controlled Radical Polymerization of a Trialkylsilyl Methacrylate by Reversible Addition-Fragmentation Chain Transfer Polymerization. *J. Polym. Sci. Part A Polym. Chem.* **2005**, *43* (22), 5680–5689.

(2) Tran, J.; Guégain, E.; Ibrahim, N.; Harrisson, S.; Nicolas, J. Efficient Synthesis of 2-Methylene-4-Phenyl-1,3-Dioxolane, a Cyclic Ketene Acetal for Controlling the NMP of Methyl Methacrylate and Conferring Tunable Degradability. *Polym. Chem.* **2016**, *7* (26), 4427–4435.

(3) Bailey, T. L.; Stubbs, C.; Murray, K.; Tomas, R. M. F.; Otten, L.; Gibson, M. I. A Synthetically Scalable Poly(Ampholyte) Which Dramatically Enhances Cellular Cryopreservation. *Biomacromolecules* **2019**, *20*, 3104–3114.

(4) Murray, K. A.; Gibson, M. I. Post-Thaw Culture and Measurement of Total Cell Recovery Is Crucial in the Evaluation of New Macromolecular Cryoprotectants. *Biomacromolecules* **2020**, *21* (7), 2864–2873.
